# Environmental Footprints of High-Speed Railway Construction in China: A Case Study of the Beijing–Tianjin Line

**DOI:** 10.3390/ijerph17010105

**Published:** 2019-12-22

**Authors:** Jianyi Lin, Shihui Cheng, Huimei Li, Dewei Yang, Tao Lin

**Affiliations:** 1 Key Lab of Urban Environment and Health, Institute of Urban Environment, Chinese Academy of Sciences, Xiamen 361021, China; jylin@iue.ac.cn (J.L.); shihuicheng@iue.ac.cn (S.C.); hmli@iue.ac.cn (H.L.); 2 University of Chinese Academy of Sciences, Beijing 100049, China; 3 School of Geographical Sciences, Southwest University, Chongqing 400715, China; younglansing@gmail.com

**Keywords:** environmental footprints, high-speed railway, construction, hybrid life cycle assessment method

## Abstract

The environmental footprints of China’s high-speed railway (HSR) have attracted much attention nationally and internationally. Although there is some research focusing on CO_2_ emissions, a comprehensive environmental impacts assessment of HSR construction is still lacking. In this study, the emissions of the Beijing–Tianjin intercity HSR line was calculated using a hybrid input–output life cycle assessment method to quantify the environmental impacts of HSR throughout its construction. The environmental footprints during the construction stage were analyzed in terms of different subsystems and sectors. The results showed that bridges contribute the largest environmental footprints at approximately 60%, followed by rail and electric multiple unit (EMU) systems. The top three sectors that contribute to pollutant emissions are the metal smelting and rolling industry, transport equipment manufacturing, and non-metallic mineral production. CO_2_ and NO_x_ are the major pollutants directly emitted by site equipment operation. More chemical oxygen demand (COD), total phosphorus (TP), total nitrogen (TN), and petroleum are emitted in EMU production than in rail construction, while NH_3_-N is emitted more in rails instead. Cd, Pb, As, and Hg are the significant pollutants in the metal smelting and rolling industry, whereas Cr, Cu, and Zn are the main heavy metal emissions in the transport equipment manufacturing sector. Heavy metals are the main types of environmental footprints in bridges, stations, and electric systems. Water pollutants are the main environmental impacts for rail and EMU systems, and the emissions of air pollutants are significant in subgrades. The production efficiency of upstream materials, desulfurization and denitration in fossil combustion, and the length of the bridge construction should be considered for an HSR under construction, in order to become environmentally friendly and sustainable.

## 1. Introduction

China’s high-speed railway (HSR) has been developing faster and at a larger scale than in any other country, thereby transforming the Chinese society and economy [1,2]. As of 2018, the HSR system of China reached a length of 29,000 km, which accounts for approximately two-thirds of the world’s total length [3]. In the same year, China’s HSR transported 2.001 billion passengers, which accounted for 60.4% of the country’s railway passenger traffic. In accordance with the Plan of Mid-to Long-term Railway Network in China, the scale of the HSR will reach 38,000 km by 2025, and an HSR grid of “eight vertical and eight horizontal” will cover most cities with a population of over 500,000 [4]. Considering the large-scale HSR network construction, an evaluation of the environmental performance of China’s HSR is an interesting initiative.

A life-cycle assessment (LCA) is a significant environmental management method for evaluating the environmental impacts of goods and services from cradle to grave, to effectively avoid the shift of environmental problems (e.g., from one region to another or a life-cycle stage to another), and mainly focuses on environmental footprints [5,6]. Environmental assessments have been applied to many types of transportation, such as road and air transport, waterway traffic, and passenger and freight railways [7,8].

Furthermore, LCAs are used to assess HSR’s environmental impacts, which can serve as guide for future HSR construction, and to reduce environmental impacts. The process analysis (PA) method, or the “bottom–up” analysis, is the first method used for the environmental impact assessment of HSRs. This method is applicable to product and activity analyses with clearly detailed processes. Many studies that applied the PA method focused on CO_2_ emissions during the different life-cycle phases of HSR. Chang and Kendall analyzed the greenhouse gas (GHG) discharge of the infrastructure construction of an HSR line in California’s HSR system, and found that most of the discharge can be attributed to the manufacturing of raw materials during the construction stage. In addition, tunnels and aerial aspect, which occupied only 15% of the infrastructure length, contributed to nearly 60% of the emissions [9]. Miyoshi and Givoni estimated the potential environmental benefit of a high-speed train in the United Kingdom, and found a relatively low potential for decreasing CO_2_ emissions [10]. Several studies also considered energy consumption aside from CO_2_ emissions. Rozycki et al. evaluated the total energy demand, material input, and CO_2_ emissions of an HSR system in Germany. The cumulative energy demand of the infrastructure only contributed 13%, whereas the energy consumption of the traction activity occupied the majority of the life cycle [11]. Åkerman analyzed a proposed HSR track in Sweden from the perspective of the life cycle. The result revealed that the reduction of the emission and energy demand can be attributed to the shifts from other transports to HSR travel [12]. Bueno et al. used a simplified tool to analyze the energy demand reduction and climate change mitigation of the HSR system in Spain [13]. In the subsequent development of environmental research, some works investigated the composite indicators of environmental impact. Chester and Horvath performed an assessment of life cycle impact (e.g., global warming and acidification potentials) to determine the relationship between the expansion of the existing infrastructure and the construction of a new HSR line. They found that the transformation from automobile trips to HSR will directly affect the environment, and the effect of GHGs will be felt within 20–30 years [14]. Yue et al. adopted the China-specific life cycle inventory database to calculate the environmental impacts in different stages. The calculation showed that the vehicle operation stage dominated most of the impact categories, and infrastructure construction contributed to a large number of organic compounds and mineral consumption in water [15]. Although the PA method enables detailed calculation, its high demand for data and time limit the method’s application to some extent.

The input–output (IO) model is a mathematical model that investigates the relationship between the inputs and outputs of various sectors of an economic system. The environmental IO analysis (IOA) method, which is used for top–down analysis, combines the environmental data with an IO table, to analyze the environmental impacts of regions or products. Banar and Özdemir adopted the life cycle cost methodologies to assess the composite environmental impact indicators of the railway passenger transportation system in Turkey. The results showed that the infrastructure accounted for 58% of the environmental impacts of the HSR line, whereas the operation stage accounted for 42% [16]. However, different products in the same sector cannot be distinguished, because this method is evaluated by sector.

The hybrid LCA method combines the advantages of PA and IOA methods to overcome PA’s difficult data collection process and IOA’s detail insufficiency. Wang and Sanders compared the energy consumption and discharge between the construction of HSRs in California and Florida using a hybrid tool, respectively. According to the high ridership prediction used to calculate energy usage and CO_2_ emissions, they found that HSR’s construction is environmentally efficient in California [17]. Jones et al. analyzed the CO_2_ and other gaseous pollutant contents of the planned HSR line from Lisbon to Porto in Portugal. They reported that the largest pollutant contributor was the operation stage, followed by the train’s manufacturing stage [18]. Similarity, there are some studies that take energy consumption into account. Chester et al. created a life cycle energy demand and emission inventory for three cities in the United States of America. They revealed that the indirect impacts of energy consumption and pollutant emissions (e.g., transportation, infrastructure, and fuel requirements) were nearly 50% larger than the vehicle’s operation [19]. Chang et al. employed the hybrid method to analyze the energy and environmental footprint of the Beijing–Shijiazhuang HSR line. The result shows that the manufacturing of steel consumed a huge amount of energy and emitted a considerable amount of CO_2_ [20]. The research on the environmental impacts of HSRs have covered different environmental pollutants in United States of America, the United Kingdom, Germany, Sweden, Spain, Portugal, Turkey, and China. However, research on the comprehensive environmental footprint indicators (covering greenhouse gases, air pollutants, water pollutants, and heavy metals) of HSR construction using a hybrid input–output life cycle assessment method is limited.

In this study, a hybrid input–output life cycle assessment method is conducted to evaluate the environmental footprints of the different subsystems of China’s HSR line. The Beijing–Tianjin intercity HSR line, which is China’s first HSR passenger line, with a designed speed of 350 km/h, was selected as a study case. Different types environmental pollutant emissions during the construction process of this line were calculated and analyzed to provide environmental guidance for future HSR construction. The remainder of this work is organized as follows. Section 1 presents the research methodology and analytical framework. Section 2 presents the case study attribute and relevant data. Section 3 contains the results of the calculations and discussions of the environmental footprints. Section 4 provides the conclusions.

## 2. Method

### 2.1. Analytical Framework

**Figure 1:**
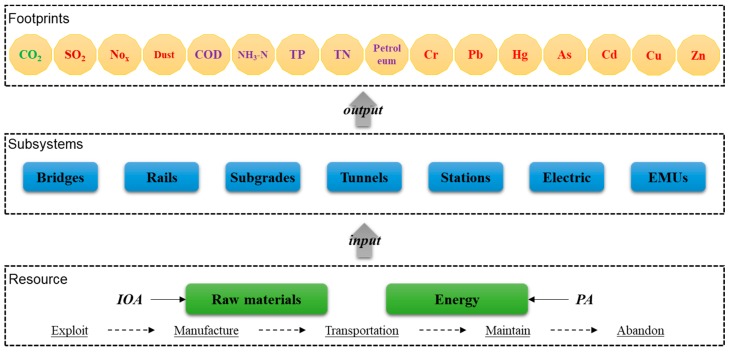
Analytical framework of the high-speed railway (HSR) system using a hybrid method. IOA—input–output analysis; PA—process analysis; COD—chemical oxygen demand (COD); TP—total phosphorous; TN—total nitrogen.

Seven subsystems are included in an HSR, such as bridges, rails, subgrades, tunnels, stations, electric system, and an electric multiple unit (EMU) system. The energy consumption and the production of building materials of all subsystems during the construction period are evaluated within the calculation boundary. The materials mainly involve reinforcement, concrete, and other infrastructure materials. Energy consumption refers to the direct utilization of energy, including the diesel and electricity used in the mechanical work during the construction stage. According to the first national pollution source census, four kinds of environmental pollutants during HSR construction are considered, including greenhouse gas (CO_2_), air pollutants (SO_2_, NO_x_, and dust), water pollutants (chemical oxygen demand (COD), NH_3_-N, total phosphorus (TP), total nitrogen (TN), and petroleum), and heavy metals (Cr, Pb, Hg, As, Cd, Cu, and Zn). A hybrid method is used to assess the life cycle environmental footprints in the stage of HSR’s construction. The top–down input–output analysis (IOA) method is adopted to calculate the emissions during the period of material production. The process analysis (PA) method is adopted to calculate the emissions from the energy use of the construction equipment. The analytical framework is shown in Figure 1.

### 2.2. Details Calculation Method

#### 2.2.1. Process Analysis

The direct environmental pollutants of energy are calculated by the PA, based on the activity level data and the corresponding emission factors. The activity level data mainly include the diesel and electricity used during the construction period. The calculation can be performed according to Equation (1):

(1) $$E_{energy}=\underset{i}{\sum }\underset{j}{\sum }\left(EF_{i,j}\times Activity_{i,j}\right)$$

where *E_energy_* denotes the pollutant discharge from energy consumption activities, *i* represents the different kinds of energy, *j* denotes the subsystem of the Beijing–Tianjin intercity HSR, *EF* denotes the pollutant emission factors of energy, and *Activity_i,j_* is the amount of energy consumption.

#### 2.2.2. Input–Output Analysis

The construction of the HSR line uses a large amount of building materials, including concrete materials (cement, sand, gravel, water-reducing agent, and slag powder) and steel products (reinforcement, steel frame, steel strand, wire, optical cable, wood, and plastic products). IOA is applied to evaluate the pollutant emissions of material production during the construction period. The environmental pollutant emissions in the material production process can be evaluated according to the following equations:

(2) $$E_{material}=F{\left(I-A\right)}^{-1}Y$$

(3) $$f=e_{k}/X_{k}$$

(4) $$y=\underset{s}{\sum }\left(1+w_{s}\right)\times P_{s}\times M_{s}$$

where *E_materia_*_l_ denotes the environmental pollutant emissions from the upstream raw material in the production process; *I* denotes the identity matrix; *A* represents the direct consumption coefficient matrix of the IO table; *(I − A)*^−1^ denotes the Leontief inverse coefficient matrix; *F* represents the matrix after diagonalizing the *f*; *f* is the environmental pollutant emission intensity matrix, which can be calculated as Equation (3); *e_k_* denotes the environmental pollutant emissions of sector *k*; *X_k_* is the economic output in the IO table of the corresponding sector; *Y* is the matrix after diagonalizing *y*; *y* is the final consumption matrix of the total material inputs, which can be calculated as Equation (4); *s* denotes the different kinds of building materials in the corresponding sectors; *w_s_* represents the wastes of the materials generated during the transportation, distribution, and utilization stages; *P_s_* denotes the piece price of material *s*; and *M_s_* denotes the whole quantity of material *s*.

## 3. Study Case

### 3.1. Case-Study Attributes

The Beijing–Tianjin intercity HSR line is a railway connecting the two major cities of Beijing and Tianjin. It is the first HSR in mainland China with a designed speed of 350 km/h, and it is also the first intercity passenger transport system constructed under the Railway Network Plan of Medium-and Long-term. The project of this line officially started in July 2005, and was opened to the public in August 2008. The whole-length of the Beijing–Tianjin intercity HSR is 120 km, of which 83.8% are bridges (100.6 km) and 16.2% are subgrades (19.4 km). The line of the Beijing–Tianjin intercity HSR has adopted the most advanced ballastless-track technology in the world, which is the first in China. The laying of the ballastless track on the entire line guarantees the high speed of the train, and can reduce dust in the air. The bridge superstructure of this line is mainly composed of pre-stressed concrete with a simple box girder. Owing to the large span of the bridges, some of them are designed as continuous beams. The line of the Beijing–Tianjin intercity HSR has five stations, namely, the Beijing South, Yizhuang, Yongle, Wuqing, and Tianjin stations, of which Yongle station is a reserved one. The line uses CRH3 and CRH2C electric vehicles with speed of 350 km/h. When the line was launched in 2008, the EMUs were organized as eight vehicles in total. After ten years of operation, the HSR has transported 250 million passengers. Hence, an investigation of the environmental footprints of the Beijing–Tianjin intercity HSR during its construction is an interesting initiative.

### 3.2. Relevant Data

The data used in the calculation include industrial energy consumption, industrial product quantity, environmental pollutant emissions, piece price of materials, input–output table, upstream raw materials of HSR, energy consumption of HSR construction, and pollutant emission factors. The data of the industrial energy consumption were sourced from the Energy Statistical Yearbook 2008 in China. The data of industrial product quantity were sourced from the Industrial Economy Statistical Yearbook 2008 in China. The environmental pollutant emissions data were based on the first national pollution source census data, which were collected in 2007 [21]. The piece price of materials and the market price of building materials were sourced from the China Price Yearbook. In this research, the IO table of China in 2007 was considered. The upstream raw materials and the energy consumption of the Beijing–Tianjin intercity HSR line during the construction were estimated based on the HSR’s design standards, construction guidelines, and past research models [22,23,24,25]. The main building materials and their corresponding sectors are shown in Table 1. The classification of sectors is based on the IO table and energy consumption sectors in China’s statistics, which are related to HSR construction. The data of the carbon emission factor for energy consumption were obtained from Energy Statistical Yearbook 2008 and the National GHG Inventory Guideline [26] in China, the data of electricity carbon emission factor in 2007 were gathered from the National Development and Reform Commission Department of Climate Change [27], and the air pollutant data of the energy consumption emission factors were sourced from the handbook on production and emission factors of industrial pollution source (Table 2).

### 3.3. Calculation Principles

According to the data availability, 2007 was selected as the calculation year, which is also within the construction period of the Beijing–Tianjin intercity HSR line between 2005 and 2008. In the process of calculating the environmental footprints of each subsystem, because tunnels were not constructed in the HSR line, only six subsystems were considered, namely bridges, rails, subgrades, stations, electric system, and an EMU system. Because the stations were not built exclusively for the Beijing–Tianjin line, they were allocated according to the route plan in 2008. The sectoral pollutant emissions in 2007 were derived from the first national pollution source census data collection [21], which excluded GHG. In addition to the pollutants involved in the census, the CO_2_ emissions in 2007, based on the activity data of energy consumption and industrial production, were also calculated. To unify the data classification and facilitate the calculation of energy consumption, the IO model was integrated into 30 sectors, according to the table of industrial sub-sector energy consumption and IO table of 42 sectors in China. Then, the eight major sectors involved during the HSR line construction period were determined and analyzed. In addition to pollutant emissions due to the production of upstream raw materials during construction, the pollutants generated by equipment operation during construction by using diesel and electricity were also considered. 

## 4. Results and Discussions

### 4.1. Greenhouse Gases

**Figure 2:**
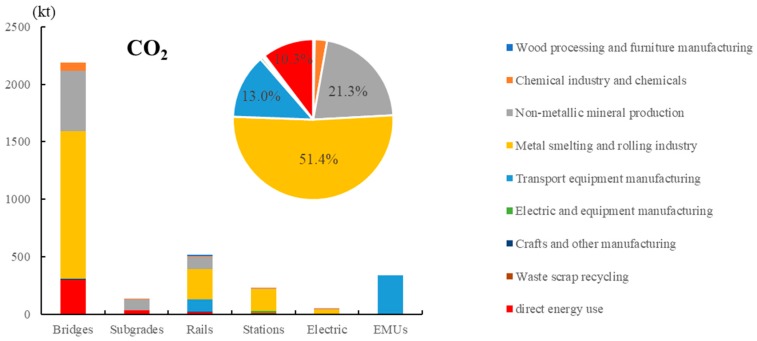
Total CO_2_ emissions of subsystems by different sources. The bar chart represents the amount of CO_2_ emitted by each subsystem; the pie chart represents the emission ratio of each sector.

Only CO_2_ was considered for greenhouse gases during the construction of the Beijing–Tianjin intercity HSR. At this stage, CO_2_ emissions were mainly derived from the upstream production of the materials and energy consumption of the construction equipment. As depicted in Figure 2, the CO_2_ emissions from the different subsystems and sectors were calculated, and the total emission caused by the construction period was 3451.7 kt. Among the different subsystems, bridges contribute the largest CO_2_ emissions in the entire stage with 2186.4 kt CO_2_, accounting for 63.3%. Considering the straight route, road settlement, and land savings, the construction of HSRs results in a large number of bridges. The Beijing–Tianjin intercity HSR line used 100.6 km of bridges to overcome the above factors, corresponding to 83.8% of the length, thereby leading to large volumes of materials and energy consumption. Rail systems rank second, with 518.6 kt CO_2_, accounting for 15.0%. The emissions from EMUs rank third, with 339.3 k*t* CO_2_, accounting for 9.8%. The remaining subgrade, station, and electric subsystems contribute 131.9, 228.8, and 46.6 kt CO_2_, respectively, accounting for a total of 11.8%.

For the emission sources, the CO_2_ emission caused by the upstream production of materials was 3094.5 kt, accounting for 89.7%. The metal smelting and rolling industry sector was the largest emitter with 1775.5 kt CO_2_, accounting for 51.4%. The non-metallic mineral production sector was the second largest emitter with 736.4 kt CO_2_, accounting for 21.3%. The third largest emitter was the transport equipment manufacturing sector with 450.2 kt CO_2_, accounting for 13.0%. A large number of metal products, organic raw materials, and earth and stones were used in the construction, leading to huge amounts of CO_2_ discharge in these sectors. Direct energy uses emit 357.2 kt CO_2_, accounting for 10.3% in the construction stage. The direct energy uses of bridges contribute the highest CO_2_ emission with 295.1 kt CO_2_, accounting for 82.6% of the entire direct energy uses. Subgrades rank second with 33.6 kt, accounting for 9.4%. The third largest contributor was rail systems with 18.0 kt CO_2_, accounting for 5.0%, and the remaining subsystems emitted a total of 10.5 kt CO_2_, accounting for 3.0%.

### 4.2. Air Pollutants

**Figure 3:**
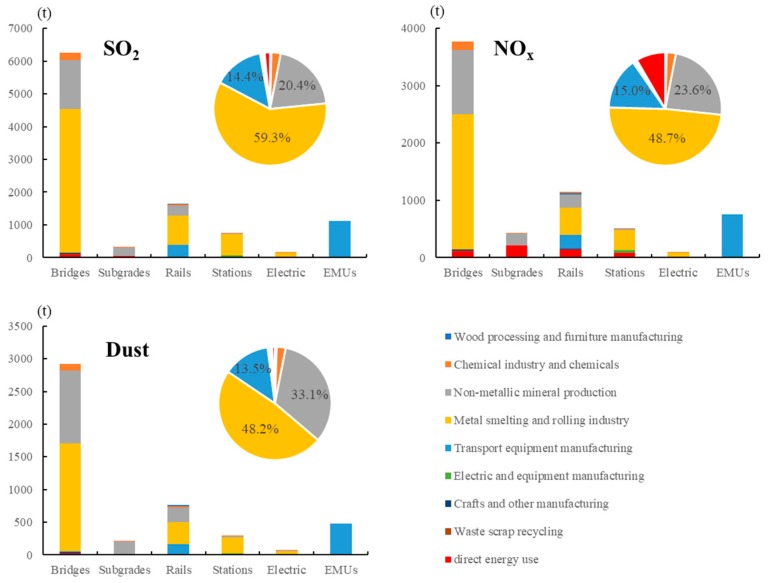
Total air pollutant emissions of subsystems by different sources. The bar chart represents the amount of air pollutants emitted by each subsystem; the pie chart represents the emission ratio of each sector.

The discharge of air pollutants from the upstream production of raw materials and the energy consumption of construction equipment are depicted in Figure 3. The amount of SO_2_ emitted by the intercity HSR during the construction stage was the largest with 10,241.7 t, followed by NO_x_ and dust with 6685.6 and 4742.9 t, respectively. The proportion of air pollutants in various subsystems and sectors was similar, which may be because gaseous pollutants are mainly derived from the combustion of fossil fuels. Among the different subsystems, bridges, rails, and EMUs were the top three emission contributors, and their proportions were similar. Bridges contributed approximately 60% of the total air pollutants, followed by rails (16%) and EMUs (11%).

For the emissions from the production of upstream materials, the metal smelting and rolling industry is the largest source of emissions, especially SO_2_, with 6069.9 t, accounting for 59.3%. This condition was because the combustion of sulfur-containing coal and petroleum was widely used in the metal smelting and rolling industry. A large amount of fuel needs to be burned during metal smelting, in which SO_2_ is emitted as the main pollutant in exhaust gas. The sector of non-metallic mineral production is the second largest contributor. In this sector, the proportion of dust emissions was the largest with 1571.2 t, accounting for 33.1%. Among the raw materials used in constructing the Beijing–Tianjin intercity HSR, the production of concrete materials, including cement and sandstone, belong to this sector, and these substances can be easily discharged as small particles during the manufacturing of non-metallic minerals. The transport equipment manufacturing sector ranked third with 1002.3 t, and the emission ratio of NO_x_ was the largest, accounting for 15.0%. Direct emission was also a main contributor to NO_x_ (8.4%), because it is emitted from fossil fuel combustion during equipment operation on construction sites, without denitration.

### 4.3. Water Pollutants

**Figure 4:**
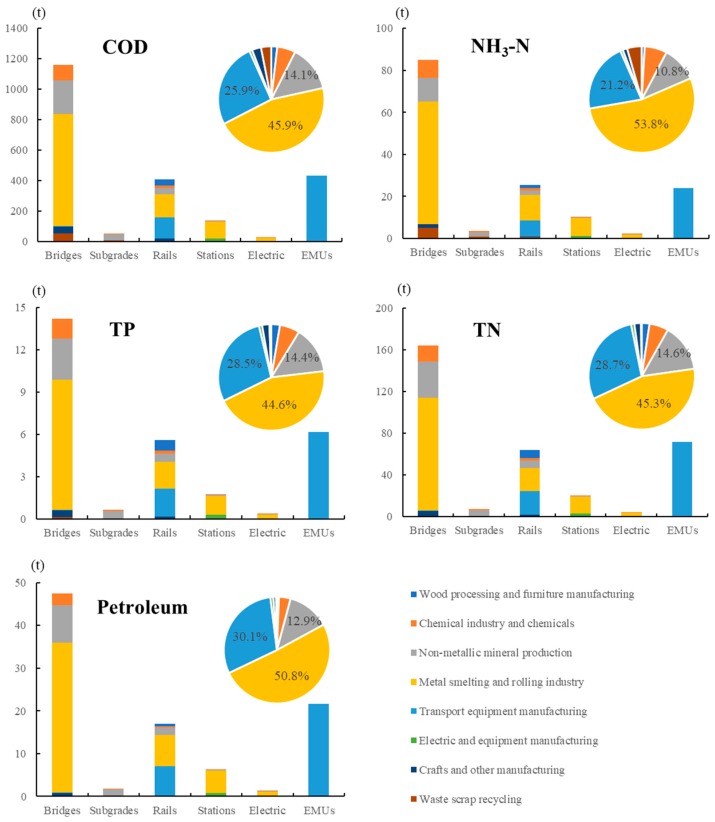
Total water pollutant emissions of subsystems by different sources. The bar chart represents the amount of water pollutants emitted by each subsystem; the pie chart represents the emission ratio of each sector.

The discharge of water pollutants during the construction of the Beijing–Tianjin intercity HSR was analyzed by comparing the emissions of five pollutants in different subsystems and sectors (Figure 4). The emission trends between the different water pollutants were relatively similar. Among the different subsystems, water pollutants, especially NH_3_-N, had the largest proportion of emissions in the bridge system, with 84.9 t emissions, accounting for 56.6%. The contribution rate of different pollutants in rails and EMUs was similar. Among the four pollutants (COD, TP, TN, and petroleum), EMU was the second largest contributor, especially petroleum, with 21.6 t, accounting for 22.7%. The rails ranked third, especially TP, with 5.6 t, accounting for 19.5%. The NH_3_-N emissions in the rails were larger than that in the EMUs, accounting for 17.0% and 16.0% of the total emissions. In the upstream production of the rails, the amount of NH_3_-N in industrial wastewater should be controlled, especially in the metal smelting and rolling industry and non-metallic mineral production sector.

From the perspective of emission sources, the five water pollutants had exactly the same sources. The metal smelting and rolling industry sector was the largest contributor, followed by the transport equipment manufacturing and the non-metallic mineral production sectors. Metal products were the main building materials in the intercity HSR, indicating that the metal smelting and rolling industry sector had the largest emission of pollutants. For the Beijing–Tianjin intercity HSR, the transport equipment manufacturing sector ranked second, and the pollutants of TP, TN, and petroleum had a relatively higher proportion than that of COD and NH_3_-N. The transport equipment manufacturing removed the oil and impurities from the parts and components, leading to the generation of sewage.

### 4.4. Heavy Metals

**Figure 5:**
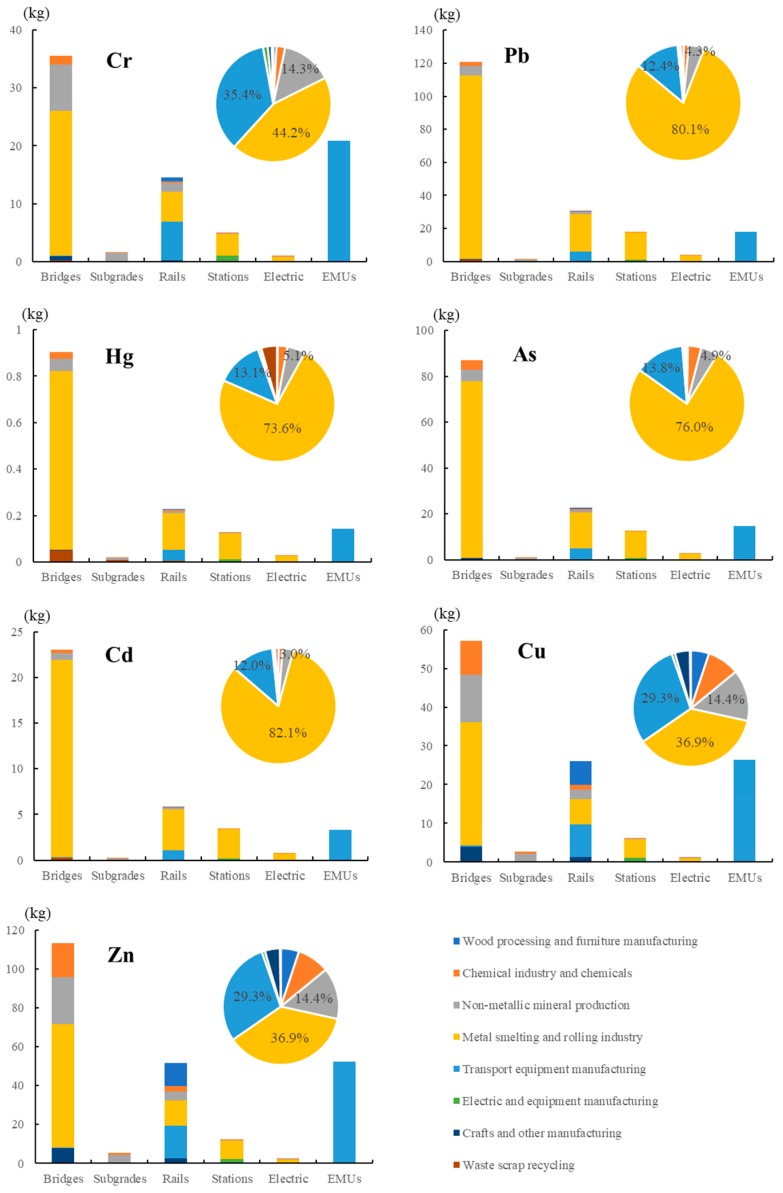
Total heavy metals emissions of subsystems by different sources. The bar chart represents the amount of heavy metals emitted by each subsystem; the pie chart represents the emission ratio of each sector.

As demonstrated in Figure 5, the emissions of different subsystems and the proportions in each source are compared. Among the different subsystems, the emissions of bridges, rails, and EMUs were the top three. The emissions during the bridge construction were the largest, because this stage consumed large amounts of engineering and building materials. The sequence of emission ratios of different heavy metals in rail and EMU systems were different. The Cr, Cu, and Zn emissions in the EMUs were significantly high, especially for Cr, with 20.9 t, accounting for 26.7%. EMU manufacturing involves a large amount of metal and cabin facilities, with Cu and Zn as the basic alloy raw materials, and the infrastructures, such as plastic, cause Cr emissions. As the sole sector of EMUs, the major heavy metal emissions in the transport equipment manufacturing sector were Cr, Cu, and Zn. The emission of other heavy metal pollutants (Pb, Hg, As, and Cd) in the EMUs was lower than that in the rail systems, representing approximately 10%. The proportion of heavy metals in the subgrade system was small, especially Pb, Hg, As, and Cd, in which the emissions in subgrades were the least among the subsystems. This condition is because the construction materials of subgrades were mainly earth and stones, and did not involve the use of metallic materials, and the amount of toxic heavy metals were low.

The analysis of the sources of seven heavy metal pollutants showed that their emissions in different sectors were consistent. The metal smelting and rolling industry sector contributed the highest emission of heavy metals, especially Cd, Pb, As, and Hg, accounting for more than 70% of the total emissions. The mining, smelting, and processing of these heavy metals allowed them to enter the atmosphere, water, and soil, thereby causing serious environmental pollution. The transport equipment manufacturing sector was the second largest emission source, especially Cr, with 34.6 t, accounting for 44.2%. The emission of Cr was mainly caused by leather preparations, the chrome plating of metal parts, and industrial rubber manufacturing. The third largest was the production of the non-metallic mineral sector, especially Cu and Zn, accounting for 29.3%. The emissions of Cu and Zn mainly came from ore mining, metal processing, and machinery manufacturing. In the initial data acquisitions (the first national census of pollution sources), the statistical caliber of Cu and Zn was the same, thereby resulting in the same final emission ratios in various industries and systems. The heavy metal pollution was mainly caused by human factors, such as mining, exhaust gas emission, sewage irrigation, and the excessive use of heavy metal products.

### 4.5. Comparation of Footprints Proportion in Each Subsystem

**Figure 6:**
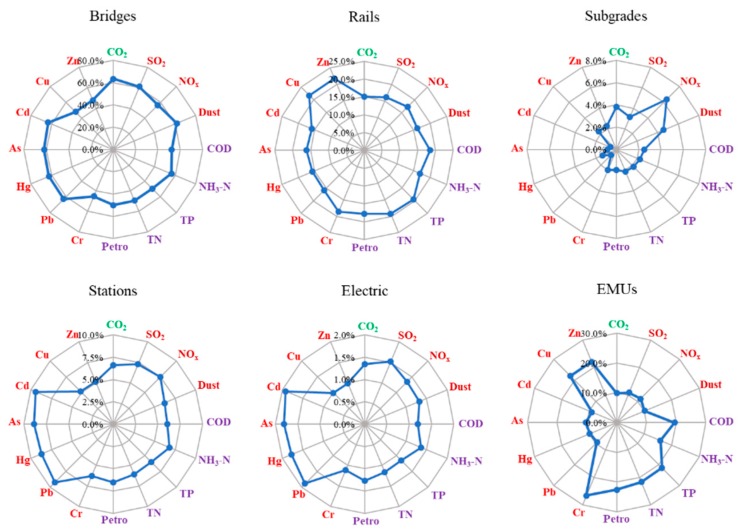
Proportions of the different environmental footprints of each subsystem.

The proportion of various pollutants in the total emissions of each subsystem was determined to identify the different environmental footprints of each of these subsystems, as shown in Figure 6. The subsystems differed greatly in construction, and their functions could be used to determine the diverse material inputs, which could also indicate differences in the emission ratios. For the bridge system, the proportion of heavy metals and air pollutants in the total emissions were significantly higher than those in the other pollutants (i.e., Pb, Hg, As, Cd, CO_2_, SO_2_, and dust). Similarly, the proportions of discharged pollutants from the stations and electric systems also showed relatively high ratios of heavy metal emissions, especially Pb, Hg, As, and Cd. The uses of metal products as building materials and electric cables can cause heavy metal emissions. For the rail system, the proportions of the pollutants were similar, but the ratios of Cu and Zn were slightly higher than the other. In contrast to these four subsystems, the ratio of heavy metal emissions in the subgrade system were significantly less than those of the other pollutants, whereas the ratio of air pollutants, such as NO_x_ and dust, were higher. The construction materials of the subgrades were mainly earthwork and stonework, which indicate the less heavy metal emissions and a higher air pollutant discharge. For the EMU system, water pollutants were notably the main source of pollutants in the system. In addition, the proportion of Cr, Cu, and Zn were high. The massive use of organic materials for EMUs led to an increase of water pollutants.

## 5. Conclusions

In this study, the environmental footprints of the Beijing–Tianjin intercity HSR line during the construction stage were evaluated using a hybrid life cycle assessment method. The environmental footprints during the construction stage of the Beijing–Tianjin intercity HSR line were analyzed in detail in terms of different subsystems and sectors. The research findings may provide environmental guidance for future HSR constructions in China, and for other countries around the world. The main research results are summarized as follows.

For the Beijing–Tianjin intercity HSR line, four types of pollutants are considered, namely, greenhouse gases (CO_2_), air pollutants (SO_2_, NO_x_, and dust), water pollutants (COD, NH_3_-N, TP, TN, and petroleum), and heavy metals (Cr, Pb, Hg, As, Cd, Cu, and Zn). Bridges are the largest contributor among the subsystems. The emissions in the rails and EMUs are included in the top three. For some water (COD, TP, TN, and petroleum) and heavy metal pollutants (Cd, Cu, and Zn), EMUs are the second largest contributor, whereas rails rank second for other types of pollutants. For the emission sources, the metal smelting and rolling industry, non-metallic mineral production, and transport equipment manufacturing sectors are the primary sources of emissions.

The metal smelting and rolling industry emits large amounts of SO_2_ because of the combustion of sulfur-containing coal and petroleum. Equipment without denitrified burned fossil fuels yield a high proportion of NO_x_ in the energy use stage. For water pollutants, the transport manufacturing sector emits more pollutants of TP, TN, and petroleum than that of COD and NH_3_-N, because of its own production process. For heavy metal emissions, the transport equipment manufacturing sector contribute large proportions of Cr, Cu, and Zn emissions, and Pb, Hg, As, and Cd are the main pollutants in the metal smelting and rolling industry of upstream material production.

The length of bridges should be considered to achieve an environment-friendly HSR during the construction stage, because of its high environmental footprints during construction and its positive effects in operation, thereby smoothing routes and saving lands. Thus, a tradeoff exists between construction and operation [28]. In the upstream production stage, the production efficiency of raw materials should be improved, especially for the three sectors of metal smelting and rolling industry, non-metallic mineral production, and transport equipment manufacturing. The desulfurization and denitration of fossil combustion should be developed in industrial production and equipment operation. The trend of sustainable development suggests that pollutant emissions should be controlled at the source that can be achieved by selecting clean raw materials. For future HSR constructions, the selection of environmentally friendly materials should be investigated in the construction industry. With regard to the production process, waste production should be reduced by improving the relevant technologies and increasing the recycling rate. The proposed hybrid method can be applied to assess the environmental footprints of other HSR lines, including the operation and maintenance stages. The research results in this paper have more reference values for nearby regions, because of the different construction conditions and material production efficiencies in other regions. More in-depth conclusions could be draw by including more of China’s HSR lines in other regions, and including more stages (construction, operation, and maintenance) in future study.

## Figures and Tables

**Table 1:** Main building materials and their corresponding sectors.

Number	Sectors	Materials
1	Wood processing and furniture manufacturing	Wood
2	Chemical industry and chemicals	Water reducer and adhesive
3	Non-metallic mineral production	Cement, sand, gravel, and asphalt
4	Metal smelting and rolling industry	Rolled steel and alloy products
5	Transport equipment manufacturing	Steel rails, rail fastener, and EMUs
6	Electric and equipment manufacturing	Electric wire and cable
7	Crafts and other manufacturing	Geotextile, plastic film, and rubber strip
8	Waste scrap recycling	Flyash and slag powder

**Table 2:** Emission factors in the stage of equipment operation during construction.

Energy Type	Related Factors	Unit	Value
Diesel	Low calorific value ^a^	KJ/kg	42,652
	Carbon content ^b^	tc/TJ	20.2
	Carbon oxidation rate ^b^	%	98
	SO_2_ emission ^c^	kg/m^3^	7.8
	NO_x_ emission ^c^	kg/m^3^	9
	Dust emission ^c^	kg/m^3^	1.8
Electricity	Carbon emission ^d^	kg/(kW·h)	0.9397

Note: ^a^ from the Energy Statistical Yearbook 2008 in China; ^b^ from the National Greenhouse Gas (GHG) Inventory Guideline in China; ^c^ from the Handbook on Production and Emission Factors of Industrial Pollution Source in China; ^d^ from the National Development and Reform Commission Department of Climate Change.

## Nomenclature

HSR	High-speed railway	PA	Process analysis
EMU	Electric multiple unit	IOA	Input–output analysis
LCA	Life cycle assessment	GHG	Greenhouse gas
CO_2_	Carbon dioxide	SO_2_	Sulfur dioxide
NO_x_	Nitrogen oxides	COD	Chemical oxygen demand
NH_3_-N	Ammonia nitrogen	TP	Total phosphorus
TN	Total nitrogen	Cr	Chromium
Pb	Plumbum	Hg	Mercury
As	Arsenic	Cd	Cadmium
Cu	Cuprum	Zn	Zinc
